# From pilot study to practice: Integrating medical students into COVID-19 contact tracing in a hospital setting

**DOI:** 10.1016/j.fhj.2024.100220

**Published:** 2024-12-09

**Authors:** Mohamed Morgan, Oscar Han, Katie Hullock, Steve Pagden, Mair Richards, Rachel Foster

**Affiliations:** a School of Medicine, University of Sheffield, UK; b Sheffield Community Contact Tracers, Sheffield, UK; c Department of Infection and Tropical Medicine, Sheffield Teaching Hospitals NHS Foundation Trust, Sheffield, UK

**Keywords:** Medical education, Pandemic, COVID-19, Contact tracing, Public health, Community engagement, Test and trace, Deprivation

## Abstract

This paper addresses a timely and significant issue in the context of the COVID-19 pandemic. Contact tracing played a crucial role in reducing transmission rates. The challenges faced in inpatient settings are valuable to explore. A pilot study involving medical students revealed gaps in engagement with the NHS Test and Trace service. An Inpatient Contact Tracing Team (IPCT) was formed to address this issue. Medical students later assisted in contact tracing.

The study was conducted from September to November 2021 and involved 305 inpatients with COVID-19.

This initiative demonstrates the benefits of involving medical students in inpatient contact tracing for public health gain and education. The students also engaged in community initiatives, addressing barriers to vaccination in disadvantaged areas. A demographic analysis revealed patterns which guided vaccine education efforts by the IPCT.

This collaboration between healthcare professionals and medical students showcases the adaptability of medical education in the face of global health challenges.

## Highlights

• A pilot study at Sheffield Teaching Hospitals revealed that 65% of COVID-19 inpatients were not engaged with the NHS Test and Trace, leading to the creation of an Inpatient Contact Tracing Team (IPCT) to address this gap.
• Medical students played a vital role in improving inpatient contact tracing, conducting face-to-face interviews and providing data analysis to help guide vaccination education efforts.
• Medical students identified significant disparities in vaccination uptake, which informed targeted vaccine education for the most deprived areas of Sheffield.
• This innovative project demonstrated the potential for medical students to contribute significantly to public health responses during pandemics.

## Introduction

The COVID-19 pandemic has led to a persistent public health crisis. One crucial strategy in managing the spread of the virus is ‘contact tracing’, which involves tracking and notifying individuals who have been in close contact with an infected person. Effective contact tracing can significantly reduce the transmission rates of infectious diseases.^1^

Contact tracing was implemented in the UK for COVID-19 on a national scale through a telephone service, ‘NHS Test and Trace’ (NHSTT), on 27 May 2020, 10 weeks after implementing a national lockdown.^2^ This centralised approach faced various challenges, such as difficulty aligning the supply of services with the demand and a lack of guidance in accessing local support services.

Infectious disease consultants at Sheffield Teaching Hospitals NHS Foundation Trust (STH) observed that NHSTT failed to identify many inpatients with COVID-19 for contact tracing. Additionally, medical education was temporarily discontinued during this time, leaving medical students underutilised in COVID-19 response activities.^3^

A pilot study was conducted in 2020, utilising medical students from the University of Sheffield supported by volunteers from Sheffield Community Contact Tracers (SCCT). This revealed that 65% of inpatients did not engage with the NHSTT service, resulting in contact tracing gaps.^4^ As a response, an Inpatient Contact Tracing Team (IPCT) consisting of NHS staff and supported by Sheffield City Council was established to address this issue at STH and became operational in July 2021. STH encompasses the Royal Hallamshire Hospital (850 beds), Northern General Hospital (1,100 beds), Jessop Wing (22 beds) and Weston Park Hospital (79 beds).^5^

This paper offers insights into the utilisation of medical students for inpatient contact tracing during the COVID-19 pandemic and their potential use in public health challenges in the future. This approach facilitates a quicker, locally targeted response, enabling early disease containment in addition to reducing the IPCT’s workload burden.

### Solution/methodology

SCCT is a volunteer-led community-based project in Sheffield. SCCT help to identify gaps in systems relating to COVID-19 and raise awareness on these issues. SCCT collaborated with the IPCT to conduct contact tracing among inpatients. Comprising retired doctors and public health specialists, SCCT also provided elective opportunities for University of Sheffield medical students. After a successful pilot in 2020, the IPCT secured funding from Sheffield City Council to operate full time. The team, consisting of three NHS workers with diverse health and social care expertise, had clinician oversight with an infectious disease consultant. In late 2021, SCCT enlisted four medical students to assist the IPCT with inpatient contact tracing and data analysis. A standardised questionnaire, developed and refined from the pilot study, aimed to enhance data collection efficiency.^4^

Developing the standardised questionnaires proved challenging as the evolving nature of the pandemic meant that the original design had to be iteratively improved to account for updated information such as additional booster doses.

The face-to-face interviews covered personal details, vaccination status, contact history with the NHSTT, potential sources of infection, visited places during the infectious period, and details of close contacts. Forms for highly suspected cases were sent to the SCCT community team. In contrast, those for confirmed cases were forwarded to the Sheffield City Council for entry into the NHSTT system, Contact Tracing and Advisory Service (CTAS). The IPCT operated across multiple sites, tracking 55–60 patients weekly.

From September to November 2021, 305 inpatients with COVID-19 were interviewed, and their data were collected and analysed. The inclusion criteria encompassed laboratory-confirmed patients and those highly suspected of COVID-19 based on typical symptoms, chest X-ray findings, or blood changes indicative of COVID-19 infection. Medical students conducted quantitative analysis on Indices of Multiple Deprivation (IMD) deciles, vaccination uptake, and age at vaccine uptake for the 305 inpatients. The anonymised data were manually entered into a spreadsheet for descriptive analysis. This revealed considerable disparities in vaccination uptake and demographics among patients,^6^ later informing the IPCT’s efforts in vaccine hesitancy education.

Before this project, there were no published data in support of the utilisation of medical students for contact tracing in the UK. Unlike previous studies in the USA, which explored telephone-based approaches, this project involved face-to-face interviews, providing a more personalised approach to contact tracing and overcoming potential technical and communication challenges.^7^

The small IPCT workforce resulted in employees doing a considerable amount of overtime to cover the large number of patients requiring contact tracing. The use of medical students provided a solution and highlighted a way to increase resources and reduce the workload of paid staff. They also were able to improve the efficiency of data collection and analysis. It is unlikely that, without the involvement of the medical students, IPCT would have been able to scrutinise the demographic and vaccine-related data of those they contact-traced and, later, often counselled about vaccine safety and uptake.

SCCT enabled the participation of medical students in community initiatives serving the most economically disadvantaged areas of Sheffield, where a significant portion of residents belong to ethnic minorities, including Roma and Pakistani communities. In these community settings, the students focussed on exploring the barriers to vaccination uptake through small group sessions and ‘Let’s Talk COVID’ street stalls. Attempts to improve vaccination uptake in these ‘seldom heard’ groups were undertaken through leaflets and poster presentations detailing information regarding the COVID-19 vaccines. This opportunity allowed students to learn about the social and health inequalities affecting these groups

Permission had to be gained to collect patient data per the hospital trust’s policies. The Caldicott guardians of STH and Sheffield City Council signed a data-sharing agreement. The UK Caldicott guardians have a statutory role and are responsible for protecting and appropriately using confidential patient details. During the pandemic, Control of Patient Information (COPI) regulations allowed patient details to be shared between STH, SCCT and the City Council under set clauses in the data sharing agreement for contact tracing. SCCT volunteers working outside STH could operate through a secure virtual private network linked to STH.

### Outcome

This project highlighted the advantages of using medical students for inpatient contact tracing. Third- and fourth-year medical students have relevant knowledge of infectious disease management and practices such as infection control, so they only require brief training. In addition to helping to control the pandemic locally, this project provided valuable educational opportunities for medical students in public health issues. It allowed them to witness the impact of health and social inequalities in COVID-19. Other hospital placements do not offer an overview of how local, community-based public health initiatives can be delivered effectively.

**Fig. 1:**
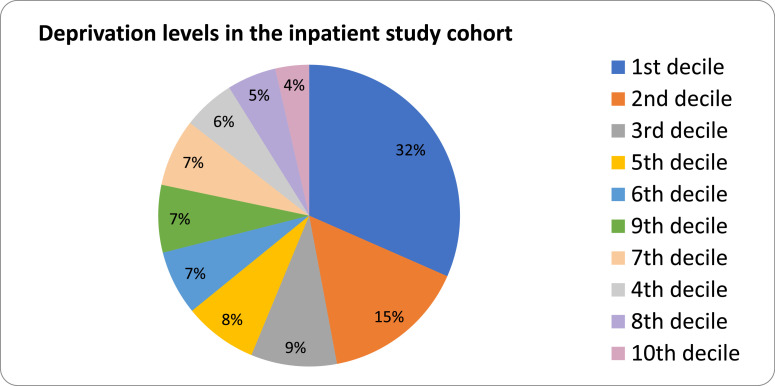
Pie chart showing deprivation levels of the inpatient study cohort. The deprivation levels are measured using IMD deciles, with ascending deciles indicating lower levels of deprivation.

Analysis by the medical students of anonymised data from the inpatient cohort revealed that almost one-third of the patients were from deprived areas of Sheffield (32%) (Fig. 1). The deprivation profile of Sheffield shows that 36.2% of residents live in neighbourhoods ranked among the 10% most deprived neighbourhoods in England. Of the 305 inpatients interviewed, 209 (68.5%) had been vaccinated.^6^

**Fig. 2:**
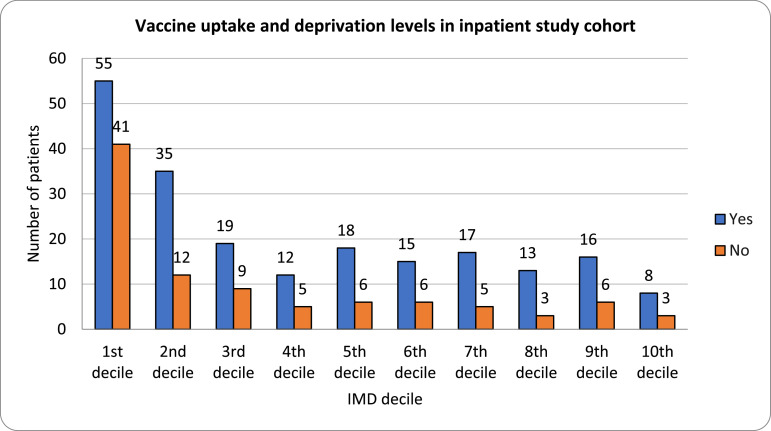
Bar chart showing vaccine uptake against deprivation levels of the inpatient study cohort.

Vaccination uptake was highest in the 8th IMD decile (81%) and lowest in the most deprived decile (44.8%) (Fig. 2).^6^ This is consistent with data from other sources in the UK.^8^

This analysis was pivotal in guiding the IPCT’s efforts in vaccine education, mainly focusing on patients hailing from the most deprived areas and those aged between 23 and 34. Identifying these demographic patterns meant that the IPCT could improve their understanding and respond to these groups’ specific concerns and barriers. The IPCT implemented culturally sensitive educational interventions and enhanced vaccine acceptance among people from these backgrounds. Furthermore, this practical, community-based project provided valuable research experience for the medical students.

The use of artificial intelligence (AI) in combating the COVID-19 pandemic is an increasingly researched field. AI could help medical students analyse much more patient data, identify transmission trends, and aid in contact tracing.^9^ Utilising AI alongside the combined efforts of the IPCT and medical students in controlling the transmission of potential infectious diseases would be valuable to explore in the future.

### Conclusion and next steps

This innovative project aimed to improve contact tracing for inpatients with COVID-19 and reduce viral transmission by combining healthcare professionals and medical students and developing suitable data collection and analysis technology.

Adequate training and supervision remain essential to ensure medical students have the necessary skills to carry out their assigned duties safely and effectively. Medical students played many roles in this initiative, including conducting interviews, data analysis and community engagement.

The demographic analysis conducted by the medical students provided crucial insights, which, in turn, guided targeted vaccine education efforts by the IPCT. This multifaceted approach, combining practical public health interventions and educational experiences, showcases the dynamic role that medical students can play in addressing complex health challenges.

While the study involved 305 COVID-19 inpatients, a larger sample size could have provided more robust findings and increased the generalisability of the results. This can be addressed through conducting a more extensive study to increase the statistical power of the findings. Furthermore, an investigation into the long-term consequences of the IPCT’s efforts, including any lasting effects on public health and healthcare systems, would provide valuable insight into the effectiveness of such a system in combating any future pandemics.

This project serves as a compelling reminder that medical students can and should be involved in both patient-facing and non-patient-facing roles in handling such unprecedented circumstances. The outbreak of future potential pandemics will inevitably affect medical education. However, learning opportunities in communication skills and health systems sciences can be garnered via other means outside of the traditional medical school curriculum, which may require updating with pandemic preparedness.^10^ Above all, it provides an invaluable lifelong personal and clinical experience for the medical students, being a part of a team that has helped tackle the pandemic from the ‘front-line’.

## Data availability

The authors confirm that the data supporting the findings of this study are available within the article [and/or] its supplementary materials.

## Ethics approval and consent to participate

This is a review of a case study and did not require ethical approval or informed consent.

## Funding

Sheffield Community Contact Tracers are funded by the Sheffield City Council.

## CRediT authorship contribution statement

**Mohamed Morgan:** Writing – review & editing, Writing – original draft, Project administration, Methodology, Formal analysis, Data curation, Conceptualization. **Oscar Han:** Writing – review & editing, Writing – original draft, Methodology, Formal analysis, Data curation, Conceptualization. **Katie Hullock:** Writing – review & editing, Writing – original draft, Project administration, Methodology, Conceptualization. **Steve Pagden:** Writing – review & editing, Supervision, Methodology, Formal analysis, Data curation, Conceptualization. **Mair Richards:** Writing – review & editing, Supervision, Formal analysis, Data curation, Conceptualization. **Rachel Foster:** Writing – review & editing, Supervision, Project administration, Methodology, Formal analysis, Data curation, Conceptualization.

## Declaration of competing interest

The authors declare that they have no known competing financial interests or personal relationships that could have appeared to influence the work reported in this paper.
